# Impact of Pre-Existing and Newly Diagnosed Atrial Fibrillation on Clinical Outcomes of Patients with Ischaemic Stroke Undergoing Endovascular Thrombectomy: Analysis of Local Data

**DOI:** 10.3390/jcm15135065

**Published:** 2026-06-29

**Authors:** Sandra Elsheikh, Greg J. Irving, Andrew M. Hill, Gregory Y. H. Lip, Azmil H. Abdul-Rahim

**Affiliations:** 1 Liverpool Centre for Cardiovascular Science at University of Liverpool, Liverpool John Moores University and Liverpool Heart & Chest Hospital, Liverpool L7 8T, UK; 2 Cardiovascular and Metabolic Medicine, Institute of Life Course and Medical Sciences, Faculty of Health and Life Sciences, University of Liverpool, Liverpool L14 3PE, UK; 3 Mersey and West Lancashire Teaching Hospitals NHS Trust, Prescot L35 5DR, UK; 4 Health Research Institute, Edge Hill University, Ormskirk L39 4QP, UK; 5 Department of Clinical Medicine, Aalborg University, 9220 Aalborg, Denmark; 6 Department of Cardiology, Lipidology and Internal Medicine, Medical University of Bialystok, 15-089 Bialystok, Poland

**Keywords:** length of stay (LOS), atrial fibrillation (AF), ischaemic stroke, endovascular thrombectomy, death

## Abstract

**Background:** Atrial fibrillation (AF) is a major risk factor for ischaemic stroke (IS) and adverse long-term outcomes. The impact of pre-existing versus newly diagnosed AF on prognosis after endovascular thrombectomy (EVT) is uncertain. **Methods:** We conducted a retrospective analysis of patients with IS admitted to Mersey and West Lancashire Teaching Hospitals NHS Trust between January 2016 and November 2023 who underwent EVT. Data were extracted from the Sentinel Stroke National Audit Programme and cross-referenced with hospital medical records. Patients were categorised into three groups based on AF status: no-AF, pre-existing AF, and new-AF. Outcomes included prolonged hospital length of stay (LOS) [≥75th percentile], poor functional outcome (modified Rankin Scale [mRS] ≥ 3 at discharge), and all-cause mortality (in-hospital, at 30-day, and at 6-month mortality). Logistic regression and Cox proportional hazards models were used for analysis. **Results:** A total of 138 patients were included (mean ± SD for age: 67.9 ± 13.9 years; 45.7% female). Hospital LOS was longest in the new-AF group (median 35.0 days [IQR: 6.0–53.0]) compared with the pre-existing AF group (18.0 days [6.0–42.0]), and the no-AF group (7.0 days [3.0–28.0]), *p* = 0.024. In adjusted logistic regression, new-AF was significantly associated with prolonged LOS (OR = 2.55, 95%CI: 1.01–6.45, *p* = 0.048) but showed no association with poor functional prognosis (*p* = 0.851). Cox regression analysis showed that AF status was not associated with mortality (*p* = 0.325). **Conclusions:** Newly diagnosed AF after stroke is associated with prolonged hospitalisation despite comparable rates of successful EVT results but had no effect on functional prognosis or risk of death.

## 1. Introduction

Atrial fibrillation (AF) is the most common arrhythmia in adults, with an estimated prevalence of 2–4% [1,2]. In the United Kingdom (UK), 1.5 million are affected by AF [3].

AF independently increases the risk of stroke [1]. Cardioembolic strokes, in which AF was reported in about 60% of cases, represent 15–30% of all ischaemic strokes [4]. Unlike other cardiovascular contributors, the attributable risk of stroke for atrial fibrillation (AF) increases with age [5]. In the Framingham Heart Study (FHS), the attributable risk of stroke for AF increased from 1.5% for those aged 50–59 years to 23.5% for those aged 80–89 years [5].

With advancement in cardiac monitoring technology, an increasing number of patients with stroke and TIA are being newly diagnosed with AF [6]. As a result, a new classification of AF in stroke patients has emerged in the recent years to include AF diagnosed after stroke and previously known AF [6]. In the UK, AF is diagnosed for the first time after stroke in 6% of cases [7]. In a meta-analysis including over 22,000 patients, newly diagnosed AF after stroke was associated with a lower prevalence of cardiovascular comorbidities and a lower risk of stroke recurrence compared to AF known prior to stroke [8].

Beyond increasing stroke risk, AF is associated with worse post-stroke outcomes [9]. In the Copenhagen Stroke Study, AF was associated with a 20% increase in the length of hospital stay, a 40% decrease in the relative chance of discharge to a patient’s own home, and a 70% increase in mortality [10]. While AF per se did not impact the stroke recovery processes in that population, its influence on those outcomes was explained by the more severe strokes seen in those with AF [10]. Similar trends were seen when assessing the mortality rates in first-time ischaemic stroke patients from the Danish Stroke Registry, where the 30-day and 1-year mortality rates were significantly higher in the AF group compared to a matched group without AF [11], but after adjusting for stroke severity, the rate was attenuated, suggesting that mortality was more likely mediated by stroke severity rather than AF [11].

Radiologically, patients with AF tend to have large cortical infarcts on computed tomography (CT), and diffuse leukoaraiosis, a marker of small vessel disease, is seen less frequently in strokes associated with AF [10]. They also have less frequent silent infarcts, lacunar infarcts, or transient ischaemic attacks (TIAs) [10].

The term “large vessel occlusion (LVO)” usually refers the occlusion of the intracranial internal carotid, basilar, proximal middle cerebral (M1), or early branch middle cerebral (M2) arteries [12]. Endovascular thrombectomy (EVT) in these patients has been proven to reduce mortality and functional dependence (assessed by measuring the modified Rankin Scale (mRS) at 90 days after stroke) [13,14]. It is recommended in addition to best medical therapy (including intravenous thrombolysis (IVT)) in all cases of anterior circulation LVO-related acute ischaemic stroke presenting within 6 hours after symptom onset [15]. In basilar artery occlusion-related strokes presenting within 6 h, EVT plus best medical therapy is recommended if the National Institutes of Health Stroke Scale (NIHSS) is ≥10 points [16].

Reperfusion after EVT is measured by the Modified Treatment In Cerebral Infarction (mTICI) scale, and technical success should be defined as mTICI 2b or higher [17]. The impact of AF on outcomes following EVT remains unclear [18]. In a pre-specified subgroup analysis of 135 patients with AF from the Multicenter Randomized Clinical trial of Endovascular treatment for Acute ischemic stroke in the Netherlands (MR CLEAN), the effect of EVT was smaller but not statistically different between the group with AF and that without [19]. On the other hand, a meta-analysis that included 6131 patients with acute ischaemic stroke (IS) revealed a significantly higher 90-day mortality in patients with AF compared to those without, despite successful reperfusion rates following EVT (defined as TICI score 2b-3) [20].

Given the heterogeneity in prior studies comparing newly diagnosed AF with known AF prior to stroke and the increasing use of EVT in our practice, we investigated the association between AF status (no-AF, pre-existing AF, and new-AF) and clinical outcomes in patients with IS treated with EVT.

## 2. Methods

### 2.1. Data Resource

This work was produced as a part of a service evaluation project to ascertain the accuracy and completeness of data provided and entered into the local Sentinel Stroke National Audit Programme (SSNAP) database and to evaluate the clinical outcomes and risk factor profile of stroke patients who had thrombectomy, comparing those with AF and those without. The SSNAP was instated to evaluate the processes and quality of care provided to stroke patients, as well as to assess the service provided by different NHS institutions for stroke patients [21]. Data are entered by all participating Trusts in a timely fashion.

The SSNAP records were used to identify all patients who met the following inclusion criteria in the period between January 2016 [when the first patient was treated with EVT] and November 2023 [the date of censoring and data collection]: 1. were admitted to Mersey and West Lancashire Teaching Hospitals NHS Trust, 2. diagnosed with stroke, and 3. received EVT. Patients with unsuccessful EVT were excluded. Baseline characteristics and outcome data were obtained from the SSNAP record. All information on SSNAP was checked against hospital records to check accuracy. When AF was diagnosed after admission with stroke, the date of diagnosis was obtained from the notes or the filed ECG. Information regarding medications and EVT outcomes were obtained from the clerking booklet and hospital notes.

### 2.2. Study Subjects and Outcomes

This study included patients with IS who underwent EVT. The initial review of the SSNAP record yielded 148 patients who were referred and reportedly transferred for the procedure.

Only patients who had a successful procedure were included. A procedure was deemed successful if the procedure itself is described as successful, if TICI 2b-3 is reported, or if flow was confirmed to be present at the time of the procedure (e.g., a patient would still be included if they went for the procedure and no thrombectomy target was identified but the flow was described to be present and only distal clots were seen). Ten patients were excluded, where the procedure was described as unsuccessful or the TICI score was reported to be 0, 1, or 2a.

**Figure 1:**
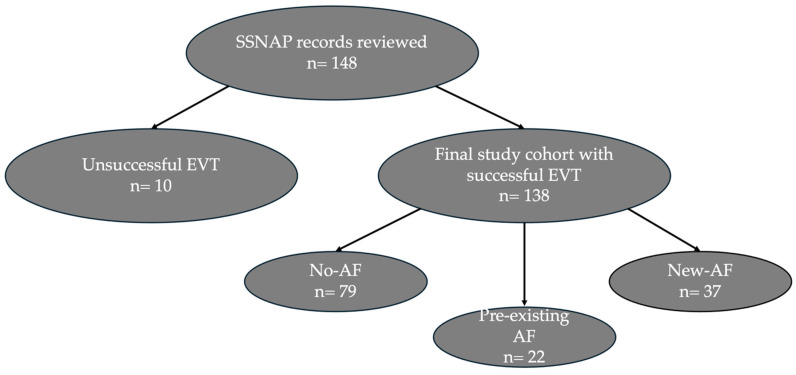
The study flowchart. SSNAP, Sentinel Stroke National Audit Programme. AF, atrial fibrillation. EVT, endovascular thrombectomy.

Then, patients were categorised into three groups based on their AF status: (1) no-AF (i.e., no history of AF before the index stroke or during their hospitalisation), (2) pre-exiting AF, and (3) new-AF (where AF was diagnosed after stroke diagnosis and during their hospitalisation). Figure 1 summarises the study flowchart.

The study outcomes included prolonged length of hospital stay (LOS) [defined as LOS greater than or equal to the 75th percentile (Q3) of the total LOS distribution in the study cohort], poor functional outcomes (mRS ≥ 3) at discharge, and mortality outcomes (in-hospital mortality, 30-day mortality, and 6-month mortality). Survival times at 30 days and 6 months were calculated from the time of patient admission until the occurrence of all-cause mortality or the end of follow-up.

### 2.3. Covariates Extraction

The following covariates were extracted from the SSNAP records and hospital admission notes for this study: age, sex, admission NIHSS score, and mRS at admission. Comorbidities such as congestive cardiac failure (CCF), hypertension, diabetes mellitus, prior ischaemic stroke, and transient ischaemic attack were also identified. Medication use at admission, including antiplatelets (aspirin and clopidogrel), anticoagulants (direct oral anticoagulants [DOACs] and warfarin), antihypertensive agents (angiotensin-converting enzyme inhibitors or angiotensin receptor blockers), beta blockers, calcium channel blockers, diuretics (bendroflumethiazide and indapamide), alpha blockers, aldosterone antagonists, and sodium-glucose cotransporter-2 (SGLT2) inhibitors, was also noted.

### 2.4. Statistical Analysis

There were no missing data. Continuous variables were assessed for normality using the Shapiro–Wilk test. Normally distributed variables were presented as the mean and standard deviation (SD) and compared between groups using ANOVA test. Non-normally distributed variables were expressed as the median and interquartile range (IQR) and compared between groups using the Mann–Whitney U test. Categorical variables were reported as counts and percentages, with differences between groups evaluated using the chi-square test or Fisher’s exact test when appropriate.

In this study, we used different statistical methods for the study outcomes. For prolonged hospital stay and poor functional outcome at discharge, we used univariate and multivariate (adjusted for age, sex, and NIHSS score) logistic regression models to calculate the odds ratio (OR) and 95% confidence interval (CI) to assess the relationship between AF status (no-AF, pre-existing AF, and new-AF, where the no-AF group served as the reference group) and their association. In addition, for the mortality outcomes, we used univariate and multivariate Cox proportional hazards models to calculate hazard ratios (HRs) and their 95% CIs to assess the associations between different AF statuses (with the no-AF group as the reference group) and all-cause mortality outcomes. Similarly, multivariate Cox proportional hazards models adjusted for age, sex, and NIHSS score were utilised. Moreover, we used Kaplan–Meier survival analysis to assess the association of AF status with 30-day and 6-month all-cause mortality. Kaplan–Meier curves were used to plot the probability of survival for patients with different AF statuses, and Log-rank tests were used to compare the differences in the survival curves among the three groups.

In this analysis, STATA (version 18; StataCorp LLC, College Station, TX, USA) and R statistical software (version 4.3.1; R Foundation for Statistical Computing, Vienna, Austria) software were used, with a two-sided *p*-value < 0.05 considered as being statistically significant.

## 3. Results

### 3.1. Baseline Characteristic

The median age differed significantly among these groups (*p*-value < 0.001), with patients with pre-existing AF being the oldest and those who were never diagnosed with AF being the youngest. For stroke severity, there was no significant difference in NIHSS score at admission among the three groups (*p*-value = 0.288), but the pre-existing AF group had a higher mRS at admission (median mRS: 1 vs. 0 in no-AF and 0 in new-AF, *p*-value < 0.001). The details are shown in Table 1.

### 3.2. Study Outcomes

The median LOS differed significantly among the groups (*p*-value = 0.024), with the longest stay observed in the new-AF group (median [IQR]: 35.0 [6.0–53.0] days), followed by the pre-existing AF group (median [IQR]: 18.0 [6.0–42.0] days), while the no-AF group had the shortest LOS (median [IQR]: 7.0 [3.0–28.0] days). The proportion of patients with prolonged LOS (≥38 days) was significantly higher in the new-AF group (43.2%) compared to the pre-existing AF (27.3%) and no-AF groups (19.0%) [*p*-value = 0.023].

Regarding functional outcomes at discharge, patients with new-AF had the highest proportion of poor functional outcomes (mRS ≥ 3) (61.0%), followed by the pre-existing AF group (64.7%) and the no-AF group (47.5%), but this difference did not reach statistical significance (*p*-value = 0.304).

**Figure 2:**
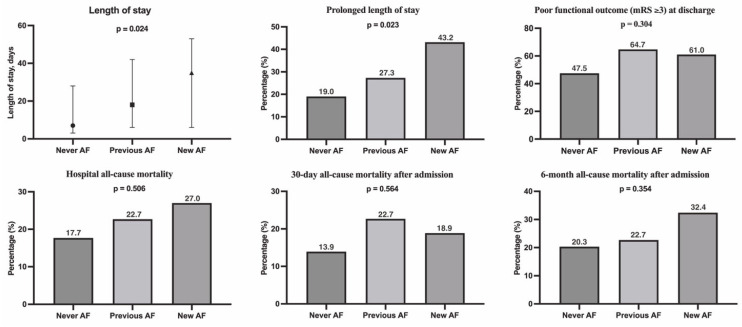
Clinical outcomes of ischaemic stroke patients stratified by atrial fibrillation status. AF, atrial fibrillation. mRS, modified Rankin Scale.

**Figure 3:**
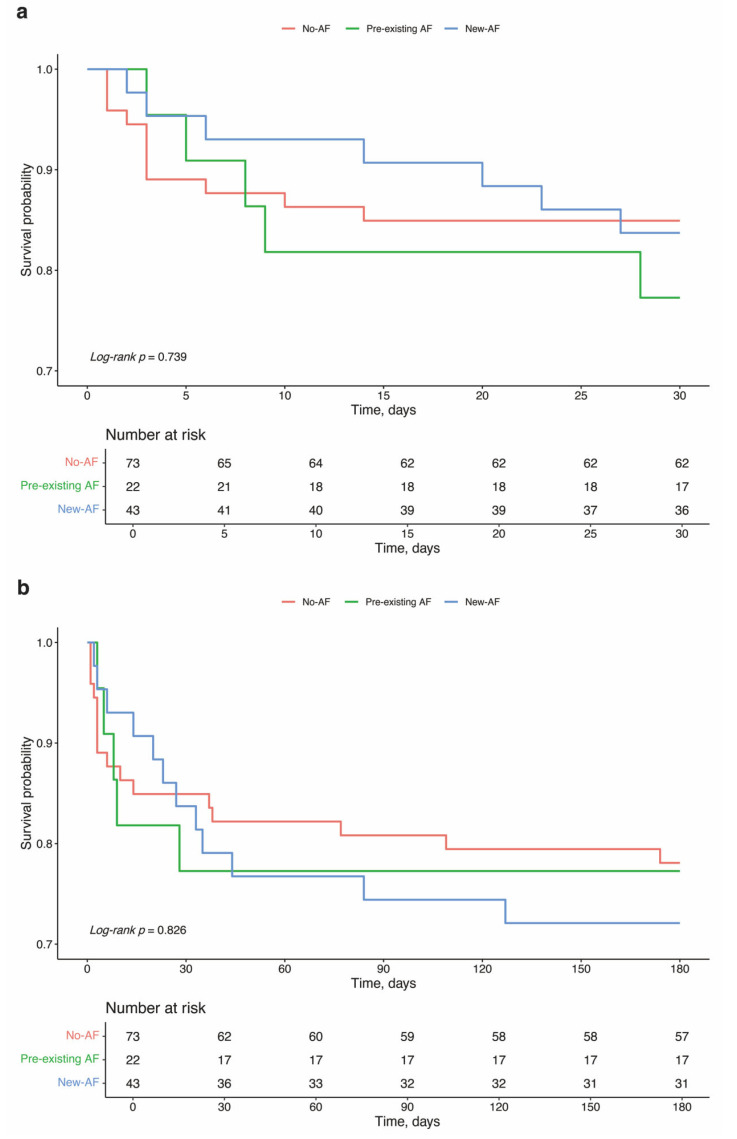
Kaplan–Meier survival curves for 30-day (**a**) and 6-month (**b**) all-cause mortality in ischaemic stroke patients with different atrial fibrillation statuses. AF, atrial fibrillation.

For mortality outcomes, the in-hospital all-cause mortality, 30-day mortality, and 6-month mortality did not differ significantly among the three groups. Numerically, the 6-month mortality was highest in the new-AF group (32.4%), followed by the pre-existing AF group (22.7%) and then the no-AF group (20.3%). The detailed study outcomes are presented in Table 2 and Figure 2. Kaplan–Meier survival analysis for 30-day (Figure 3a) and 6-month (Figure 3b) all-cause mortality revealed no significant differences in survival probabilities among the three groups (30-day mortality: log-rank *p*-value = 0.739; 6-month mortality: log-rank *p*-value = 0.826).

### 3.3. Logistic Regression Analysis of AF Status, Length of Stay, and Functional Outcomes

From Table 3, in the unadjusted model, patients with new-onset AF had 3.25-fold higher odds of prolonged LOS (OR = 3.25, 95% CI: 1.38–7.68, *p*-value = 0.007) compared to those who were never diagnosed with AF. After adjusting for potential confounders, this association remained statistically significant (OR = 2.55, 95% CI: 1.01–6.45, *p*-value = 0.048). In contrast, pre-existing AF was not significantly associated with prolonged LOS in either the unadjusted (OR = 1.60, *p*-value = 0.400) or adjusted model (OR = 0.85, *p*-value = 0.802).

Regarding functional outcomes, new-onset AF was not associated with poor functional status (mRS ≥ 3) at discharge in the unadjusted model (OR = 1.70, 95% CI: 0.72–4.05, *p*-value = 0.228). After adjustment for confounders, this association was not statistically significant (OR = 1.67, 95% CI: 0.60–4.61, *p*-value = 0.325). Prior AF was not significantly associated with poor functional outcomes in both the unadjusted and adjusted models (*p*-value = 0.215 and *p*-value = 0.764, respectively).

### 3.4. Cox Proportional Hazards Analysis of AF Status and Mortality Outcomes

To assess the relationship between AF status and mortality outcomes, we performed Cox proportional hazards analyses (Supplementary Table S1). In both the unadjusted and adjusted models, neither pre-existing AF nor new-AF during hospitalisation was significantly associated with an increased risk of in-hospital all-cause mortality, 30-day mortality, or 6-month mortality compared with patients who were never diagnosed with AF. For previous AF, the adjusted HRs were 0.78 (95% CI: 0.25–2.37, *p*-value = 0.657) for in-hospital mortality, 1.50 (95% CI: 0.45–4.97, *p*-value = 0.504) for 30-day mortality, and 0.81 (95% CI: 0.28–2.41, *p*-value = 0.711) for 6-month mortality; all were not statistically significant. Similarly, the adjusted HRs for new-AF during hospitalisation were, respectively, in-hospital mortality = 0.94 (95% CI: 0.41–2.18, *p*-value = 0.888), 30-day mortality = 1.29 (95% CI: 0.47–3.52, *p*-value = 0.617), and 6-month mortality = 1.35 (95% CI: 0.62–2.94, *p*-value = 0.451), showing no significant association.

## 4. Discussion

This study explored the association between AF status (no-AF, previous AF, and new-AF) and clinical outcomes in patients with ischaemic stroke who underwent successful EVT. We found that patients with pre-existing AF were older and had a higher prevalence of CCF and hypertension, with greater use of beta-blockers and DOACs. Stroke severity at admission did not significantly differ among the study groups, but patients with pre-existing AF had a higher baseline mRS, suggesting poorer pre-stroke functional status. Regarding hospitalisation outcomes, new-AF was associated with the longest LOS, with a significantly higher proportion experiencing prolonged hospitalisation (≥38 days) compared to the other groups, and this association remained significant after adjusting for confounders. Functional outcomes at discharge were numerically worse in the new-AF group, but statistical significance was not reached. In terms of mortality outcomes, in-hospital, 30-day, and 6-month all-cause mortality outcomes did not statistically significantly differ among these groups, although numerically, the 6-month mortality was highest in the new-AF group.

In our study, the incidence of AF diagnosed during the hospital stay was 26.8%. In comparison to the Copenhagen Stroke study, which included 1197 acute stroke patients treated on a stroke unit from the admission time to the end of rehabilitation, 18% were newly diagnosed with AF on admission [10]. In the Framingham Study, where 5070 members, aged 30 to 62 years with no prior cardiovascular disease, were followed up for 38 years, the incidence of AF diagnosed newly on admission was 3.2% [22]. The higher incidence of “new” AF in our study might be because of the specific subgroup of stroke patients included, and all our patients had EVT for LVO strokes, which have been well linked to AF [18].

Whether AF newly diagnosed after stroke is a different entity to pre-existing AF has been widely studied. One study suggested that in patients with IS, pre-existing AF and “new’ AF diagnosed after stroke may have different pathophysiological mechanisms. Pre-existing AF is thought to be of cardiogenic origin and more associated with cardiac comorbidities, while AF diagnosed after stroke is thought to develop because of the neurogenic and autonomic cardiac changes induced by the stroke [23]. The brain–heart axis also plays an important role in the complex bidirectional relationship between stroke and arrhythmias [24]. Stroke-induced brain injury may activate afferent pathways that transmit proinflammatory signals via the hypothalamus and brainstem, thereby amplifying the peripheral inflammatory response and promoting neurogenic inflammation [24]. This increases catecholamine release at the myocardial nerve endings increasing the sympathetic tone and causing arrhythmias [24]. Over time, the increased catecholamine exposure can also result in cardiomyocyte necrosis, hypertrophy, and fibrosis [24]. Another study showed that AF newly diagnosed after stroke is likely a precipitant for it rather than a consequence of it [22]. In our study, the two groups had similar in-hospital mortality rates, but the 6-month mortality rates were numerically higher in the new-AF group. Although our study did not observe an increase in short-term mortality, this may be related to the anticoagulation management of some of the patients, the type of stroke (i.e., size and territory of the infarct), and the short follow-up period. In addition, prior anticoagulation for patients known to have AF prior to their stroke was associated with less severe strokes, smaller infarct size, and reduced risk of haemorrhagic transformation [25]. Hence, compared with new-onset AF, patients with pre-existing AF in our cohort may have been treated with anticoagulation, which may explain to some extent the difference in prognosis. Moreover, the high risk of bleeding after stroke may limit the implementation of early anticoagulation leading to adverse clinical outcomes in patients with new diagnosis of AF, in whom anticoagulation strategies may need to be individually assessed to balance stroke recurrence and bleeding risk. On the other hand, while management in patients with pre-existing AF anticoagulation may have been an established strategy, attention still needs to be paid to the adaptation of anticoagulation after stroke, especially in the short term after EVT. Future studies should further explore management strategies for patients with new-onset AF and pre-existing AF after stroke and EVT, including the optimal timing of anticoagulation, the safety of antiplatelet-and-anticoagulation combination, and the assessment of long-term stroke recurrence and bleeding risk, to guide more precise and individualised therapy plans.

In the present study, we found that patients with new-AF had longer hospital stays and performed worse in terms of functional outcomes than patients with pre-existing AF or those who were never diagnosed with AF, although no significant difference in short-term mortality was seen. This suggests that patients may face additional adverse effects due to the development of new-onset AF after EVT. Therefore, in terms of clinical management of patients after EVT, enhanced rhythm monitoring in such patients is essential. Wearable smartwatches and implantable cardiac monitors may help to improve the detection rate of AF [26,27] and thus optimise subsequent treatment decisions. As prior anticoagulation for patients known to have AF prior to their stroke was associated with less severe strokes, smaller infarct size, and reduced risk of haemorrhagic transformation [25], anticoagulation strategies for new-AF need to be individually assessed to balance stroke recurrence and bleeding risk.

### Strengths and Limitations

Our study has several strengths. First, we investigated the occurrence of postoperative AF and its prognostic implications in stroke patients undergoing EVT, a high-risk group that has rarely been analysed separately in previous studies. Thus, this study fills a gap in the field. Second, this study not only distinguished between the patient groups with new-AF and those with previous AF but also compared the differences in clinical outcomes after IS between these two groups, making the findings more clinically instructive. In addition, we evaluated multiple outcomes, including prolonged LOS, poor functional outcome (using mRS score), and all-cause mortality at different time points (in hospital, at 30 days, and at 6 months). The combined analysis of these metrics comprehensively reflects the impact of AF status on the prognosis of patients after IS.

However, our study has several limitations. First, this was a retrospective analysis and, hence, susceptible to recall bias and missing information due to incomplete records. Second, due to difficulty controlling for all potential confounding factors (such as use of certain medications), it is difficult to establish causation. Third, while all consecutive patients who were admitted to Whiston Hospital with acute stroke and received EVT were included, the sample size was small, making it difficult to detect or amplify differences between the study groups. Fourth, the results also have limited external validity and cannot be generalised. Fifth, as our dataset was primarily based on the locally collected SSNAP records, we only had access to certain information with regards to patients’ comorbidity state and outcomes, e.g., information regarding the reasons for not being on anticoagulation in patients with pre-existing AF was not available. Sixth, the new-AF was identified based on in-hospital monitoring; however, intermittent or short-duration AF episodes might have been missed, particularly in patients without continuous ECG monitoring. This could result in misclassification and an underestimation of the association between new-AF and outcomes. Seventh, although we adjusted for NIHSS at admission, differences in stroke severity, infarct size, and treatment approaches (e.g., variability in mechanical thrombectomy technique or timing of anticoagulation initiation) might still have influenced outcomes, introducing potential bias. Eighth, our study focused on in-hospital, 30-day, and 6-month outcomes, but longer-term consequences of AF, such as stroke recurrence, AF progression, and anticoagulation-related complications, were not assessed. Lastly, in patients with severe stroke who died early, new-AF may not have been detected in the relatively short period of illness. This could have led to potential bias in assessing its prognostic significance.

## 5. Conclusions

Our study demonstrated that patients with new-AF were more likely to experience longer hospital stays and demonstrate lower functional independence at discharge. Prospective studies are required to better understand the mechanisms connecting AF, thrombectomy outcomes, and long-term prognosis.

## Figures and Tables

**Table 1:** Baseline characteristics stratified by atrial fibrillation status.

Characteristic	All	No-AF	Pre-Existing AF	New-AF	*p*-Value
N	138	79	22	37	
Age, years		66.0 (54.0, 73.0)	80.5 (72.0, 86.0)	73.0 (65.0, 79.0)	<0.001
Female, *n* (%)	63 (45.7)	30 (38.0)	12 (54.6)	21 (56.8)	0.110
NIHSS at admission, score	19.0 (14.0, 23.0)	18.0 (13.0, 22.0)	21.0 (15.0, 24.0)	19.0 (17.0, 24.0)	0.288
mRS at admission, scale	0.0 (0.0, 1.0)	0.0 (0.0, 0.0)	1.0 (0.0, 2.0)	0.0 (0.0, 1.0)	<0.001
Comorbidities, *n* (%)					
Congestive cardiac failure, *n* (%)	12 (8.7)	3 (3.8)	8 (36.4)	1 (2.7)	<0.001
Hypertension, *n* (%)	72 (52.2)	33 (41.8)	13 (59.1)	26 (70.3)	0.013
Diabetes mellitus, *n* (%)	18 (13.0)	8 (10.1)	3 (13.6)	7 (18.9)	0.385
Prior ischaemic stroke, *n* (%)	8 (5.8)	3 (3.8)	3 (13.6)	2 (5.4)	0.191
Prior TIA, *n* (%)	8 (5.8)	2 (2.5)	4 (18.2)	2 (5.4)	0.025
Medications, *n* (%)					
Aspirin, *n* (%)	18 (13.0)	9 (11.4)	2 (9.1)	7 (18.9)	0.501
Clopidogrel, *n* (%)	8 (5.8)	4 (5.1)	0 (0.0)	4 (10.8)	0.261
DOAC, *n* (%)	8 (5.8)	0 (0.0)	7 (31.8)	1 (2.7)	<0.001
Warfarin, *n* (%)	6 (4.4)	2 (2.5)	4 (18.2)	0 (0.0)	0.011
ACEI/ARB, *n* (%)	50 (36.2)	23(29.1)	11 (50.0)	16 (43.2)	0.115
Statins, *n* (%)	54 (39.1)	24 (30.4)	14 (63.6)	16 (43.2)	0.015
Beta blockers, *n* (%)	43 (31.2)	14 (17.7)	16 (72.7)	13 (35.1)	<0.001
CCB, *n* (%)	22 (15.9)	10 (12.7)	2 (9.1)	10 (27.3)	0.121
Bendroflumethiazide, *n* (%)	8 (5.8)	3 (3.8)	1 (4.6)	4 (10.8)	0.344
Indapamide, *n* (%)	4 (2.9)	2 (2.5)	0 (0.0)	2 (5.4)	0.624
Alpha blockers, *n* (%)	5 (3.6)	2 (2.5)	1 (4.6)	2 (5.4)	0.555
Aldosterone antagonists, *n* (%)	6 (4.4)	1 (1.3)	4 (18.2)	1 (2.7)	0.008
SGL2 inhibitors, *n* (%)	2 (1.5)	0 (0.0)	0 (0.0)	2 (5.4)	0.095

Abbreviations: ACEI, angiotensin-converting enzyme inhibitor; AF, atrial fibrillation; ARB, angiotensin ii receptor blocker; CCB, calcium channel blocker; DOAC, direct oral anticoagulant; mRS, modified Rankin Scale; NIHSS, National Institutes of Health Stroke Scale; SGL2 inhibitors, sodium-glucose cotransporter-2 inhibitors; TIA, transient ischaemic attack.

**Table 2:** Outcomes of patients stratified by atrial fibrillation status.

Outcome	All	No-AF	Pre-Existing AF	New-AF	*p*-Value
Length of stay, days	9.5 (4.0, 38.0)	7.0 (3.0, 28.0)	18.0 (6.0, 42.0)	35.0 (6.0, 53.0)	0.024
Prolonged length of stay, *n* (%)					0.023
Length of stay < 38 days	101 (73.2)	64 (81.0)	16 (72.7)	21 (56.8)	
Length of stay ≥ 38 days	37 (26.8)	15 (19.0)	6 (27.3)	16 (43.2)	
^†^ Poor functional outcome (mRS ≥ 3) at discharge, *n* (%)	59 (54.1)	28 (47.5)	11 (64.7)	20 (61.0)	0.304
Hospital all-cause mortality, *n* (%)	29 (21.0)	14 (17.7)	5 (22.7)	10 (27.0)	0.506
30-day all-cause mortality after admission, *n* (%)	23 (16.7)	11 (13.9)	5 (22.7)	7 (18.9)	0.564
6-month all-cause mortality after admission, *n* (%)	33 (23.9)	16 (20.3)	5 (22.7)	12 (32.4)	0.354

^†^ A total 29 patients were not included as they passed away. Abbreviations: AF, atrial fibrillation; mRS, modified Rankin Scale.

**Table 3:** Logistic analysis of atrial fibrillation status with prolonged length of stay and functional outcomes.

		Unadjusted		Adjusted	
Outcomes	AF Status	OR (95% CI)	*p*-Value	OR (95% CI)	*p*-Value
Prolonged length of stay					
	No-AF	Reference		Reference	
	Pre-existing AF	1.60 (0.54, 4.78)	0.400	0.85 (0.25, 2.92)	0.802
	New-AF	3.25 (1.38, 7.68)	0.007	2.55 (1.01, 6.45)	0.048
^†^ Poor functional outcome (mRS ≥ 3) at discharge	No-AF	Reference		Reference	
	Pre-existing AF	2.03 (0.66, 6.21)	0.215	1.23 (0.33, 4.62)	0.764
	New-AF	1.70 (0.72, 4.05)	0.228	1.10 (0.42, 2.88)	0.851

^†^ A total of 29 patients were not included as they passed away. Model was adjusted for age, sex, and National Institutes of Health Stroke Scale. Abbreviations: AF, atrial fibrillation; CI, confidence interval; mRS, modified Rankin Scale; OR, odds ratio.

## Data Availability

The data underlying this study are not publicly available due to patient confidentiality and institutional governance restrictions. Data may be available from the corresponding author on reasonable request, subject to approval from Mersey and West Lancashire Teaching Hospitals NHS Trust.

## Supplementary Materials

The following supporting information can be downloaded at: https://www.mdpi.com/article/10.3390/jcm15135065/s1, Table S1: Cox proportional hazards analysis investigating the association between of AF status and mortality outcomes.
