# Supplementary material for: Impact of Pre-Existing and Newly Diagnosed Atrial Fibrillation on Clinical Outcomes of Patients with Ischaemic Stroke Undergoing Endovascular Thrombectomy: Analysis of Local Data
Source: J Clin Med. 2026 Jun 29;15(13):5065. doi: 10.3390/jcm15135065 (PMC13362718; doi:10.3390/jcm15135065)
Supplement: Supplementary file 1 [file jcm-15-05065-s001.zip › jcm-4311884-supplementary.pdf]

Impact of Pre-existing and Newly Diagnosed Atrial Fibrillation on Clinical Outcomes of Patients with Ischaemic Stroke Undergoing Endovascular Thrombectomy: Analysis of Local Data

Supplementary Table S1. Cox proportional hazards analysis investigating the association between of AF status and mortality outcomes

		Unadjusted		Adjusted	
Outcomes	AF status	HR (95% CI)	p	HR (95% CI)	p
In-hospital all-cause mortality					
	No-AF	Reference		Reference	
	Pre-existing AF	0.99 (0.35, 2.76)	0.981	0.78 (0.25, 2.37)	0.657
	New-AF	0.99 (0.44, 2.25)	0.983	0.94 (0.41, 2.18)	0.888
30-day all-cause mortality after admission					
	No-AF	Reference		Reference	
	Pre-existing AF	1.63 (0.57, 4.68)	0.367	1.50 (0.45, 4.97)	0.504
	New-AF	1.33 (0.51, 3.42)	0.559	1.29 (0.47, 3.52)	0.617
6-month all-cause mortality after admission	No-AF	Reference		Reference	
	Pre-existing AF	1.15 (0.42, 3.13)	0.789	0.81 (0.28, 2.41)	0.711
	New-AF	1.63 (0.77, 3.46)	0.198	1.35 (0.62, 2.94)	0.451

Model was adjusted for age, sex, and National Institutes of Health Stroke Scale.
Abbreviations: *AF,* atrial fibrillation; *CI,* confidence interval; *HR,* hazard ratio.
